# Mitochondrial Dysfunction and Parkinson’s Disease—Near-Infrared Photobiomodulation as a Potential Therapeutic Strategy

**DOI:** 10.3389/fnagi.2020.00089

**Published:** 2020-04-03

**Authors:** Aaron Song Chuan Foo, Tuck Wah Soong, Tseng Tsai Yeo, Kah-Leong Lim

**Affiliations:** ^1^Department of Physiology, National University of Singapore, Singapore, Singapore; ^2^Division of Neurosurgery, Department of Surgery, University Surgical Cluster, National University Hospital, Singapore, Singapore; ^3^Lee Kong Chian School of Medicine, Nanyang Technological University, Singapore, Singapore; ^4^Department of Research, National Neuroscience Institute, Singapore, Singapore

**Keywords:** Parkinson’s disease, mitochondria dysfunction, neurodegeneration, energy dysregulation, near infrared

## Abstract

As the main driver of energy production in eukaryotes, mitochondria are invariably implicated in disorders of cellular bioenergetics. Given that dopaminergic neurons affected in Parkinson’s disease (PD) are particularly susceptible to energy fluctuations by their high basal energy demand, it is not surprising to note that mitochondrial dysfunction has emerged as a compelling candidate underlying PD. A recent approach towards forestalling dopaminergic neurodegeneration in PD involves near-infrared (NIR) photobiomodulation (PBM), which is thought to enhance mitochondrial function of stimulated cells through augmenting the activity of cytochrome C oxidase. Notwithstanding this, our understanding of the neuroprotective mechanism of PBM remains far from complete. For example, studies focusing on the effects of PBM on gene transcription are limited, and the mechanism through which PBM exerts its effects on distant sites (i.e., its “abscopal effect”) remains unclear. Also, the clinical application of NIR in PD proves to be challenging. Efficacious delivery of NIR light to the substantia nigra pars compacta (SNpc), the primary site of disease pathology in PD, is fraught with technical challenges. Concerted efforts focused on understanding the biological effects of PBM and improving the efficiency of intracranial NIR delivery are therefore essential for its successful clinical translation. Nonetheless, PBM represents a potential novel therapy for PD. In this review, we provide an update on the role of mitochondrial dysfunction in PD and how PBM may help mitigate the neurodegenerative process. We also discussed clinical translation aspects of this treatment modality using intracranially implanted NIR delivery devices.

## Introduction

Parkinson’s disease (PD) is one of the most prevalent neurodegenerative disorders worldwide. Its incidence rises steeply above the age of 65, with one in every 50 people above the age of 80 being diagnosed with PD (Titova and Chaudhuri, 2018). From 1990 to 2016, the number of PD patients across the globe more than doubled from 2.5 million to 6.1 million. At the current pace of growth, this number is projected to hit 12 million by 2040 (Dorsey et al., 2018), making PD the most rapidly accruing neurological disease worldwide. Epidemiological data from developed countries (United Kingdom, United States, Singapore, Brazil) estimates the cost of each PD patient to be between USD 5,835–10,349 annually. As the natural course of PD is slow and progressive, PD patients continue to live for many years in a state of dependence (Hoehn and Yahr, 1998). Productivity losses, the need for a permanent carer, nursing home costs and hospitalization fees constitute the main expenses for upkeeping this group of patients (Findley, 2007; Zhao et al., 2013; Bovolenta et al., 2017). In response to the projected surge of PD, several groups have advocated urgent action to address this impending “pandemic” (Dorsey et al., 2018).

The reason for progressive disability in PD patients is the dysregulation of neural circuits in the basal ganglia that are responsible for movement control. One of the characteristic features of PD is the selective degeneration of dopaminergic neurons in the substantia nigra pars compacta (SNpc), which exerts a profound influence on muscle tone, ambulation, and limb movement through its effects on the basal ganglia motor loop. A hypo-dopaminergic state, as exemplified in PD, manifests clinically with “lead-pipe” rigidity of the affected limbs, “bradykinesia” i.e., generalized slowing of movement, and a distinctive 4–6 Hz “pill-rolling” tremor which characteristically affects the distal part of one’s upper extremity and spreads proximally before involving the contralateral side. Histologically, affected neurons exhibit an exuberance of intracytoplasmic inclusions containing misfolded α-synuclein proteinaceous fibrils—a neuropathological hallmark of PD known as “Lewy bodies” (Spillantini et al., 1997). Post-mortem analysis of PD brains has led to the proposal that Lewy body pathology is propagative, with the lower brainstem, upper brainstem and cerebral hemispheres being involved at successive stages of the disease (Del Tredici and Braak, 2016). In recent times there has also been an increasing body of evidence implicating the gut-brain axis in the spread of Lewy pathology, with alterations in the gut microbiome being the proposed mechanism for initiation of α-synucleinopathy (Braak et al., 2003a,b; Phillips et al., 2008; Fasano et al., 2013; Tan et al., 2014; Hasegawa et al., 2015; Kim et al., 2019; Yang et al., 2019). Intriguingly, not all neurons that demonstrate Lewy body accumulation undergo significant degeneration. Similarly, at any one stage, only a select group of neurons in each diseased region demonstrates the predilection for α-synuclein fibril aggregation (Surmeier and Schumacker, 2013). These peculiar observations suggest that the most severely affected neuronal groups ought to possess traits that confer increased vulnerability to α-synucleinopathy-mediated insults.

## Bioenergetics and Selective Neuronal Vulnerability in PD

**Figure 1:**
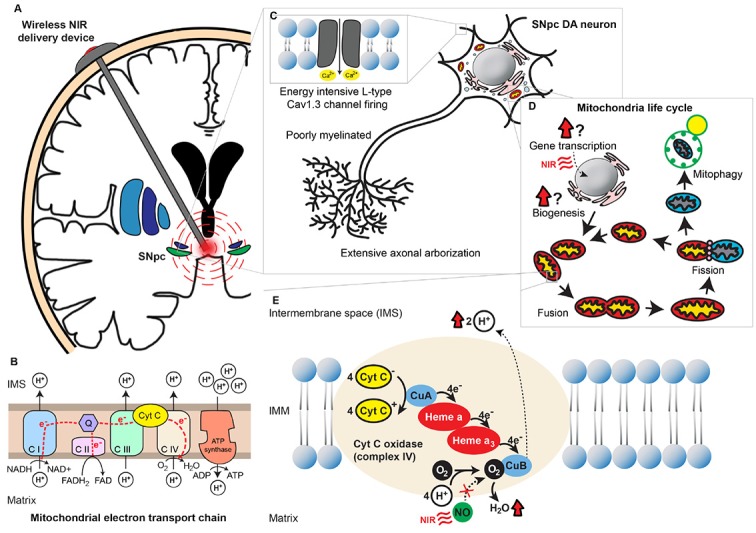
Near-infrared (NIR) stimulation and its potential neuroprotective role in Parkinson’s disease (PD). **(A)** A wirelessly powered intracranial device implanted in the human brain for delivery of NIR PBM to the SNpc. **(B)** Mitochondrial oxidative phosphorylation chain consisting of 5 different transmembrane protein complexes on the IMM that comprise the electron transport chain (ETC). The ETC pumps electrons derived from the Krebs cycle across the IMM into the intermembranous space through the actions of NADH dehydrogenase (complex I) and succinate dehydrogenase (complex II). The electrons are subsequently transferred to ubiquinone, which is reduced to ubiquinol upon electron receival. Cytochrome bc1 complex (complex III) then transfers these ubiquinol bound electrons to cytochrome C, which in turn binds to cytochrome C oxidase (complex IV) and facilitates the final electron transfer to oxygen. This transfer of electrons is coupled with the outward movement of protons across the IMM, establishing a transmembrane electrochemical gradient. Potential energy stored in the transmembrane gradient [proton motive force Δ*P*_m_] is utilized by adenosine triphosphate (ATP) synthetase (complex V) to drive the production of ATP from ADP and phosphate, which is coupled to the inward flow of protons across the IMM. **(C)** Energy-demanding features of SNpc dopaminergic neurons. These neurons possess extensive axonal arborization, long and poorly myelinated axonal projections, as well as L-type Cav1.3 channel-mediated pacemaker-type firing. Cumulatively these properties deplete the reserve capacity of these neurons to respond to an energy crisis. **(D)** The mitochondria life cycle depicting the processes of biogenesis, fusion, fission, and degradation. The effects of NIR on these processes remain uncertain. **(E)** An illustration of mitochondrial complex IV, COX, with its heme and copper centers. Electrons are transferred from cytochrome C to O_2_ *via* a series of redox reactions involving these centers. Binding of endogenous NO to O_2_’s binding site on CuB and prevents the final electron transfer step in oxidative phosphorylation. NIR PBM is purported to act *via* photodissociation of NO from O_2_’s binding site, releasing the NO’s inhibition on COX and thereby enhancing mitochondrial energy production. Abbreviations: ATP, Adenosine triphosphate; C I, Complex I; C II, Complex II; C III, Complex III; C IV, Complex IV; C V, Complex V; CuA, Copper A; CuB, Copper B; Cyt C, Cytochrome C; DA, Dopaminergic; IMM, Inner mitochondrial membrane; IMS, Intermembrane space; NIR, Near-infrared; NO, Nitric oxide; O_2_, Oxygen; Q, Coenzyme Q; PBM, Photobiomodulation; SNpc, Substantia nigra pars compacta.

Neurons consume much larger amounts of energy compared to other cell types as the maintenance of resting membrane potential is an extremely energy-intensive process (Purves et al., 2018). Not surprisingly, neuronal groups with increased vulnerability in PD possess characteristics that contribute to a greater energy burden (Figure 1). Neurons with their cell bodies in the dorsal motor nucleus of the vagus (DMV), the medullary reticular nuclei, the pontine raphe nuclei, the locus coeruleus (LC), the pedunculopontine nucleus (PPN) and the SNpc share similar morphological features—long, thin and poorly myelinated axons with extensive terminal branching (Sulzer and Surmeier, 2013). These structural attributes impose substantial challenges on neuronal metabolism. First, the excessively arborized axonal tree implies a multi-fold increase in the number of synaptic terminals, which places a strain on vesicular recycling and mitochondrial trafficking machinery (Liang et al., 2007). Second, the lack of myelination gives rise to multiple leak points along the axon, increasing the burden of resting membrane potential maintenance. Atop the inefficiencies of their morphological characteristics, several of these neuronal groups also possess burdensome physiological phenotypes. Unlike the majority of neurons whose activity is triggered by the opening of voltage-gated ion channels in response to membrane depolarization, these neurons fire action potentials even in the absence of external stimulation due to their innate pace-making abilities (Williams et al., 1984; Chan and Chan, 1989; Travagli et al., 1991; Zhang et al., 2012; Cooper et al., 2015). Autonomous pace-maker dependent firing prolongs the amount of time these neurons spend in the depolarized state, which commensurately increases the amount of energy needed by adenosine triphosphate (ATP)-dependent ion pumps to maintain resting membrane potential. Finally, some of these neuronal groups (e.g., SNpc dopaminergic neurons, DMV cholinergic neurons) rely on Cav1.3 L-type calcium channel-mediated pacemaker activity for sustained autonomous firing. Cav1.3 generated currents create broad action potentials, implying slow opening/closing kinetics which lead to larger amounts of Ca^2+^ influx. In comparison with Na^+^ currents, Ca^2+^ currents are far more energy-intensive to maintain as the gradient of [intracellular Ca^2+^]:[extracellular Ca^2+^] is 2,000 times that of [intracellular Na^+^]:[extracellular Na^+^] (Surmeier and Schumacker, 2013). Ca^2+^ dependent firing also strains intracellular Ca^2+^ buffering mechanisms and predisposes the neuron to excitotoxicity. Cumulatively, the energy-intensive properties of the above neuronal groups lower their capacity to cope with an energy crisis. This translates into increased neuronal vulnerability when exposed to further stresses, such as α-synucleinopathy in PD.

## Mitochondria—The Energy Powerhouses of the Cell

The fact that energy-intensive neurons have increased vulnerability in PD suggests that some of the underlying disease processes may be linked to impairments in cellular energy production. This lends further weight to the hypothesis that disruptions in mitochondrial function constitute some of the key pathogenic processes in PD.

Mitochondria are believed to have originated from the fusion of an oxidative α-propeobacterium with a pre-eukaryotic cell 1.5–2 billion years ago (Margulis, 1975; Zimmer, 2009; Richards and Archibald, 2011). This union achieved a synergistic effect, with the resultant organism being capable of harnessing oxidative phosphorylation—a mechanism of energy production 15 times more efficient than the process of anerobic glycolysis utilized by unicellular organisms (Kadenbach, 2012). The ability to cope with higher energy demands enabled these hybrids to grow to larger dimensions and paved the way for their evolution into pluricellular organisms—the present-day eukaryotes. The oxidative phosphorylation system (Figure 1) comprises of the mitochondrial electron transport chain (ETC) and ATP synthase, all of which are transmembrane proteins located on the inner mitochondrial membrane (IMM). Complex IV of the ETC, cytochrome C oxidase (COX), is thought to be the enzyme that catalyzes the rate-limiting step in the chain of electron transfers (Villani and Attardi, 1997). This property is unique to mammalian cells and is not observed in isolated mitochondria. It is believed that COX has a regulatory role in cellular energy production, which varies with tissue-type and metabolic demands. The presence of tissue-specific isoforms of cytochrome C and COX in mammals lends strength to this theory (Pierron et al., 2012). Neuronal energy levels fluctuate in sync with their frequency of action potential firing. Unsurprisingly, it has been demonstrated *in vitro* that COX expression closely mirrors neuronal activity (Liang et al., 2006b). Being a bi-genomically encoded enzyme with a total of 13 subunits, transcription of both nuclear and mitochondrial DNA (mtDNA) is necessary for COX expression (Kadenbach et al., 2000). This process is mediated *via* transcription factors nuclear respiratory factors 1 and 2 (NRF1 and NRF2), which have been identified as promoters of COX expression in times of increased cellular energy demand (Ongwijitwat and Wong-Riley, 2005).

In addition to the regulation of oxidative phosphorylation, maintaining healthy mitochondrial volumes is also essential for efficient energy production in cells. The intracellular mitochondrial network is constantly pruned by the processes of biogenesis, fusion, fission, and degradation. These processes constitute the mitochondrial life cycle, which takes place every 5–20 min (Ferree and Shirihai, 2012). Under physiological conditions, the events in the mitochondrial life cycle are in dynamic equilibrium, maintaining a stable mass of intracellular mitochondria. If cellular metabolic requirements are altered, the mitochondrial network undergoes remodeling to accommodate the changes in energy demand (Ferree and Shirihai, 2012). The various stages in the mitochondrial life cycle are illustrated in Figure 1. Excellent reviews on the various aspects of the mitochondrial life cycle have been written elsewhere and we will therefore not elaborate on this topic in the present article (Hyde et al., 2010; Youle and Narendra, 2011; Ferree and Shirihai, 2012).

## Linking Mitochondrial Dysfunction With PD

The mitochondrial theory of PD is based on observations in which disease processes impairing oxidative phosphorylation, mitochondrial biogenesis or mitophagy manifest as phenotypes that share common Parkinsonian features. The first link between PD and mitochondrial dysfunction was conceived by Langston (2017) after observing rapidly developing Parkinsonian features in intravenous drug abusers who were exposed to 1-methyl-4-phenyl-1,2,3,4-tetrahydropyridine (MPTP), a toxin whose metabolite 1-methyl-4-phenylpyridinium (MPP+) inhibits mitochondrial complex I (Ramsay et al., 1986, 1987; Langston, 2017). Once in the central nervous system, MPP+ is taken up by the dopamine active transporter and induces the selective degeneration of SNpc dopaminergic neurons. This phenomenon has been successfully reproduced in MPTP-treated rodents and nonhuman primates, popularizing these animal models for PD research (Dauer and Przedborski, 2003; Konnova and Swanberg, 2018). Complementing the findings in MPTP, rotenone, a pesticide that inhibits mitochondrial complex I, produced similar PD-like locomotor deficits in treated animals. Despite it having widespread uptake in the central nervous system, the toxic effects of rotenone are most pronounced in SNpc dopaminergic neurons, reinforcing the belief that neurons with greater susceptibility to energy perturbances would be affected the most (Betarbet et al., 2000). Further evidence is gleaned from epidemiological studies, which identified rotenone-exposed agrarian populations to be at greater risk of developing PD (Dhillon et al., 2008; Tanner et al., 2011).

Genetic mutations in PD patients further cement the link between mitochondrial dysfunction and PD. Mutations in the *PINK1* and *PRKN* (formerly *PARK2*) genes, which encode the mitophagy-related proteins PINK1 and Parkin, respectively, result in autosomal recessively inherited, early-onset forms of PD (Kitada et al., 1998; Valente et al., 2004). Phenotypes of Parkin-type and PINK1-type early-onset PD are strikingly similar, implicating not just individual gene mutations but the entire mitochondrial quality control machinery in the pathogenesis of PD (Bruggemann and Klein, 1993; Schneider and Klein, 1993). Concomitantly, mutations in mtDNA have been identified in sporadic PD patients. The number of these somatic mutations accumulate with age, mirroring the increased incidence of PD in older subjects (Corral-Debrinski et al., 1992). As several ETC proteins require bi-genomic transcription involving both nuclear and mtDNA (Hermann et al., 2012), an accumulation of mtDNA mutations would likely impair mitochondrial oxidative phosphorylation capacity. Interestingly, post-mortem studies in aged patients depict a higher prevalence of mtDNA mutations in the SNpc compared to other parts of the brain, regardless of whether these patients had PD or not (Soong et al., 1992; Bender et al., 2006, 2008). Whilst the reasons underlying why mtDNA mutations have a predilection for accumulating in SNpc neurons remain elusive, these findings align aging, PD and mitochondrial bioenergetic failure in a unifying pathogenic process beginning with strained mitochondrial function due to age-related genomic instability, subsequent bioenergetic failure of the most vulnerable neuronal groups, and finally, neuronal loss leading to the disease phenotype. This belief is further substantiated by age-related Parkinsonism observed in the MitoPark mouse, a transgenic mouse model with conditional knockout of mitochondrial transcription factor A (Tfam) in dopaminergic neurons. MitoPark mice are born healthy but develop progressive degeneration of SNpc dopaminergic neurons and motor retardation as they grow older, mimicking the natural course of PD in humans (Sterky et al., 2011). On the whole, evidence supporting mitochondrial dysfunction as one of the key pathogenic processes in PD is compelling and promotes acceptance of PD as a disease of bioenergetic failure.

## Near-Infrared Photobiomodulation as a Strategy to Promote Mitochondrial Function

As the mitochondrial theory of PD gains prominence, therapeutics aimed at mitochondrial modulation are also garnering interest in the PD community. Near-infrared (NIR) light therapy, also known as “photobiomodulation” (PBM), is a treatment modality that has been under investigation for many ailments since the 1960 s. Discovery of the therapeutic properties of NIR light was rather accidental—in an experiment where photodynamic therapy was delivered to tumor cells in rodents, the intensity of laser administered was mistakenly reduced, and whilst this failed to achieve the intended tumoricidal effects of the experiment, the attenuated laser therapy successfully promoted superficial wound healing in the treated animals (Mester et al., 1971). This accidental finding revealed the therapeutic effects of low-intensity laser therapy and prompted further investigation of its mechanism of action.

PBM works on the principle that light-sensitive molecules in the body known as chromophores are excited by photonic stimulation. It is now known that hemoglobin, myoglobin, and COX are the only 3 chromophores in mammalian tissue capable of absorbing light in the NIR range (600–900 nm wavelength). Passarella and Karu (2014) were the first to discover that one of the main NIR-sensitive chromophores was associated with mitochondria. In the early experiments conducted by Passarella et al. (1984, 1988), irradiation of rat liver mitochondria with low dose Helium-Neon laser light of 632.8 nm wavelength enhanced oxygen consumption, increased membrane potential, promoted ATP generation and encouraged protein synthesis. In their attempts to identify the molecule responsible for these effects, Karu et al. (1996) experimented with NIR PBM on HeLa cells, believing that by identifying the wavelength(s) at which delivered light produced the greatest cellular response they could determine the peak absorption spectrum of the target molecule and hence uncover its identity. Interestingly, their study revealed peak DNA/RNA synthesis and cellular adhesive levels at multiple wavelengths within the NIR range—620 nm, 680 nm, 760 nm and 820 nm (Karu et al., 1996; Karu, 1999). These wavelengths corresponded with excitation energies required for metal-ligand and d-d electron transfers in copper (Cu) complexes and prompted the search for a photo-acceptor with multiple Cu centers. As mitochondrial complex IV COX possessed two redox-active Cu sites (Cu_A_ and Cu_B_) within its core, it emerged as the apparent candidate responsible for the effects of NIR.

Mitochondrial cytochrome C undergoes oxidation by COX and in turn facilitates the reduction of O_2_ to water as per the chemical equation below (Sarti et al., 2012).

$$4\;cytochrome\;c^{2+}+O_{2}+8\;H_{\text{in}}^{+}\to 4\;cytochrome\;c^{3+}+2H_{2}O+4H_{\text{out}}^{+}$$

The transfer of electrons from cytochrome C to O_2_ is coupled with the expulsion of 4 protons from the mitochondrial matrix across the IMM, contributing to the transmembrane electrochemical gradient that drives ATP synthesis. Nitric oxide (NO), a molecule known for its vasodilatory properties *in vivo*, competes with O_2_ for its binding site on the Cu_B_ center of COX (Hamblin, 2018). By binding noncovalently to CU_B_, NO inhibits the activity of COX and hence impairs the process of oxidative phosphorylation. It is surmised that under physiological conditions, the inhibitory effect of NO may have a role in the regulation of energy production. To maximize the efficiency of resource utilization, the body reduces O_2_ consumption in tissues whose metabolism is low (e.g., keratinocytes) to ensure that demand in energy-intensive tissues (e.g., neurons, hepatocytes and cardiomyocytes) can be met (Lane, 2006). The prevailing theory underpinning PBM is that NIR enhances mitochondrial energy production by causing photodissociation of NO from its binding site on COX, which facilitates O_2_ binding and electron transfer (Hamblin, 2018). In a series of noteworthy experiments, Wong-Riley et al. (2001, 2005) investigated the mechanism through which PBM modulated energy production in rodent visual cortex neuronal cultures. Being post-mitotic cells with energy demands that are largely reflective of their spiking activity, neurons are ideal candidates for bioenergetic studies. Wong-Riley et al. (2001, 2005) demonstrated that while PBM with 670 nm wavelength light was capable of fully reversing the effects of tetrodotoxin (TTX) induced sodium (Na^+^) channel blockade on neuronal COX activity (Wong-Riley et al., 2001, 2005), its effect was attenuated significantly when TTX was substituted with potassium cyanide (KCN), a potent COX inhibitor (Wong-Riley et al., 2005). These results strongly suggested that the beneficial effects of PBM were at least partially mediated through enhancing mitochondrial COX activity. Further supplementation of the above results by findings demonstrating that PBM augmented neuronal ATP levels in the presence of toxin treatment further supported its role as an enhancer of mitochondrial energy production (Wong-Riley et al., 2005; Ying et al., 2008).

Notwithstanding these claims, there have been several alternative hypotheses refuting the mitochondrial COX theory of PBM in recent years. For example, Lima et al. (2019) demonstrated increased cellular proliferation after the administration of NIR PBM in cell lines and animal models with deficient COX expression. An alternative mechanism to augmenting COX activity has also been suggested by another study showing that enhanced mitochondrial energy production is the effect of a reduction in intra-mitochondrial water viscosity induced by NIR (Sommer, 2019). It is believed that the viscosity of the thin layer of water molecules on the inner side of the IMM, termed the interfacial water layer (IWL), influences the driving rate of ATP synthase—the enzyme that catalyzes ATP production in the mitochondria. ATP generation is facilitated by the rotatory motion of the ATP synthase machinery, which is in turn propelled by the diffusion of protons across IMM as a result of an established transmembrane electrochemical gradient. NIR-mediated reduction in IWL viscosity decreases the friction opposing ATP synthase rotation and results in “smoother” turning of the ATP synthase machinery. This theory is supported by the fact that cellular ATP level increases are immediate after PBM stimulation (Quirk et al., 2016), suggesting that the intracellular changes mediating elevated ATP production are alterations in physical properties rather than chemical processes, as the latter tends to occur with a slight time lag (Sommer, 2019). Yet another group suggested that the photo-absorbent metabolite pyropheophorbide-a (P-a) of dietary chlorophyl could facilitate light-driven energy production processes in animals (Xu et al., 2014). Xu et al. (2014) first examined whether P-a associates with the mitochondria after chlorophyl ingestion by looking at fluorescence emission levels of P-a from mammalian mitochondrial fragments. Elevated levels of 675 nm fluorescence emission characteristics of P-a were noticed after a certain concentration of mitochondrial fragments was amassed, suggesting the possibility of P-a being bound to mitochondrial proteins. This finding was followed up with experiments measuring ATP levels in isolated mammalian mitochondria treated with P-a and/or 670 nm wavelength NIR light. In the subsequent experiment, ATP levels only increased in groups where P-a and NIR light were co-administered, and not in those in which P-a or NIR were given in isolation. This observation was further supported by *in vivo* demonstration in a *Caenorhabditis elegans* model that lifespan, respiration rate, and ATP levels increased significantly when both P-a and NIR were administered concomitantly (Xu et al., 2014). Given the multiplicity of these competing theories, it may be that NIR exerts its modulatory effects through several mechanisms instead of one.

Intriguingly, the beneficial effects of NIR PBM follow a biphasic dose-response curve which adheres closely to the Arndt-Schulz principle (Huang et al., 2009; Sharma et al., 2011; Xuan et al., 2016). At very low doses of stimulation, the effects of PBM are negligible. As the dose of PBM is increased its effects are correspondingly amplified until a peak is observed, oftentimes at an intensity that is still considered relatively low. Further increments of intensity beyond this level only serve to elicit inhibitory or harmful effects in treated specimens. This property of NIR has led to the belief that the PBM delivers its benefits by acting as a gentle stressor of cells, which triggers the activation of intrinsic cellular stress response machinery. Part of this response involves the activation of transcription factors and secondary signaling pathways, which can explain the sustained effects of PBM even after a single treatment session (Liang et al., 2006a). The nuclear transcription factor NF-kB is upregulated in response to PBM-induced elevation of intracellular reactive oxygen species (ROS) and leads to activation of several anti-apoptotic, antioxidant and pro-proliferation downstream processes (de Freitas and Hamblin, 2016). Whilst low-stress levels achieve beneficial outcomes for cells, overzealous NIR stimulation leads to pathological levels of ROS production, which initiates a self-propagating vicious cycle that begets more ROS release, cellular damage, and activation of intracellular apoptotic pathways (Zorov et al., 2006). It seems like cellular tolerance for ROS depends on the capacity of mitochondrial superoxide dismutase (MnSOD) to cope with the partitioning of superoxide (O^−^) radicals, and once a critical threshold is breached any benefits associated with NIR are nullified (Stone and Yang, 2006; Sies, 2017).

There are some reports that NIR PBM also promotes neurogenesis and synaptogenesis atop its neuroprotective properties. An increase in neuro-progenitor proliferation and synaptogenesis markers have been observed in the dentate gyrus and subventricular zone after PBM stimulation in rodents (Xuan et al., 2013, 2014, 2015). Furthermore, Johnstone et al. (2014) proposed that in addition to its local cellular effects PBM also elicits a systemic type of response that is immunomodulatory (Rochkind et al., 1989; Johnstone et al., 2015). This arises from clinical observations in which the local application of NIR can elicit positive effects at distant sites in the body. The exact mechanisms underpinning this phenomenon are still unknown, but is believed to involve the release of trophic factors (e.g., brain-derived neurotrophic factor), downregulation of pro-inflammatory cytokines and upregulation of anti-inflammatory cytokines in the body (Braverman et al., 1989; Johnstone et al., 2014). One such effector molecule is PPARγ, a nuclear receptor involved in the production of anti-inflammatory heat shock protein 70 (HSP 70), which is increased after NIR treatment (de Lima et al., 2013; Croasdell et al., 2015). The distant effects of NIR has been likened to the “abscopal effect” observed in radiotherapy of metastatic cancer, where local treatment of a tumor not only leads to its shrinkage but also a diminution of metastases at distant sites (Johnstone et al., 2015).

## PBM as a Potential Therapeutic Solution for PD

The concept of using PBM as a therapeutic approach in PD is particularly enticing for several reasons: first and foremostly, the mechanisms underpinning both disease and intervention converge on the common subject of mitochondrial bioenergetics. That PD is gaining recognition as a disease of bioenergetic failure makes PBM, which is focused on enhancing cellular energy production, a befitting remedy for this disease. This matchup is further complemented with neuroinflammation being another proposed pathogenic process in PD and NIR purported to have both local and systemic immunomodulatory effects (Johnstone et al., 2015). Long term PBM administration has been shown to limit astrogliosis, a consequence of neuroinflammation that recruits exuberant infiltrates of astrocytes and microglia to form glial scars (El Massri et al., 2016, 2018; O’Brien and Austin, 2019). Further supporting this claim are observed reductions of immunogenic cytokine levels after PBM administration (Byrnes et al., 2005; Muili et al., 2012). Being able to address two of the key pathogenic processes in PD makes NIR an attractive candidate for clinical translation amidst the list of experimental therapeutics targeted at neurodegeneration. Further promoting the feasibility of NIR is the absence of adverse events associated with its usage, which suggests a robust safety profile. To date, there have been no acute adverse effects reported with NIR PBM delivered in doses within the determined therapeutic ranges [4–30 J/cm^2^; 5–50 mW/cm^2^], both in short and long term animal and patient studies (Desmet et al., 2006; Ilic et al., 2006; Zivin et al., 2009; McCarthy et al., 2010; Rojas and Gonzalez-Lima, 2011; Quirk et al., 2012a; Moro et al., 2017; Hamilton et al., 2019). With negligible side effects and the potential for immense benefits, the favorable risk: reward ratio of NIR light therapy significantly raises its feasibility for clinical application. Lastly, NIR PBM has demonstrated the potential to be neuroprotective, which is unprecedented in PD therapeutics. At present, there are no treatments that can forestall the neurodegenerative process in PD. The principle modality of PD treatment is drug replacement therapy, which is targeted at artificially restoring central nervous system dopamine levels either by inhibition of dopamine metabolizing enzymes or replacement by levodopa. The effects of drug treatment eventually wear off as the disease progresses into its later stages and the non-physiological pattern of dopamine replacement in the central nervous system triggers untoward dyskinetic movements in PD patients which can prove to be extremely debilitating. Once the undesirable side effects of medical therapy become intolerable, advanced PD treatments such as deep brain stimulation, continuous subcutaneous apomorphine infusions, and intra-jejunal levodopa-carbidopa intestinal gel (LCIG) are offered to patients. Although being able to effectively address medication failure and levodopa-induced dyskinesia, these treatments remain extremely costly and are unable to confer protection against the neurodegenerative process in PD. The anticipated “pandemic” of PD patients in the coming years and its associated escalation in healthcare costs herald the need for new therapies focusing on neuroprotection in the disease’s early stages.

A few groups have examined the neuroprotective effects of NIR PBM in *in vitro* PD models (Table 1). For example, Ying et al. (2008) and Liang et al. (2008) have demonstrated reduced cell death, increased ATP production and elevated COX activity after delivery of pulsed 670 nm wavelength light in PD neurotoxin-treated rodent primary neuronal cultures, A30P over-expressing neuroblastoma cell lines and PD cybrid cells with damaged mtDNA (Zorov et al., 2006; Trimmer et al., 2009; Quirk et al., 2012b). Several principles guiding optimal delivery of NIR stimulation were derived from such *in vitro* studies. First, NIR seems to work best with regimes requiring pre-treatment. The neuroprotective effects of PBM are most accentuated when its administration starts before toxin treatment of neurons and continues throughout toxin exposure (Wong-Riley et al., 2005; Liang et al., 2006b; Ying et al., 2008). Nonetheless, this phenomenon was not necessarily observed in *in vivo* models, as evidenced by the absence of benefit in NIR pre-treated *vis-á-vis* NIR post-treated mice which received MPTP (Reinhart et al., 2016). The benefits of pre-treatment with NIR can allude to ischemic preconditioning in which episodes of tolerable ischemia mitigates damage from a serious insult that follows, such as in stroke or peripheral arterial disease. Second, the optimal dose of NIR for neuronal stimulation is fluence and radiance values of 4–30 J/cm^2^ and 50 mW/cm^2^, respectively (Wong-Riley et al., 2005; Liang et al., 2006b; Ying et al., 2008). Keeping to these parameters would avert light toxicity from NIR, in keeping with the biphasic dose-response of NIR stimulation as per the Arndt–Schultz Law. Lastly, lights in the NIR spectrum that produce the greatest stimulatory effects in neurons are those at 670 nm and 830 nm wavelengths (Wong-Riley et al., 2005); 670 nm being relatively close to the peak absorption spectrum of oxidized Cu_B_ (Karu, 1999). The successful optimization studies performed in neuronal cultures paved the way for PBM testing in animal models, in which subsequent rodent and non-human primate experiments often used the above treatment paradigms as their benchmark.

**Table 1:** *In vitro* studies in PD models.

Reference	Source	NIR Treatment parameters	Model	Toxin treatment	Effects
Wong-Riley et al. (2001)	LED	670 nm at 50 mW/cm^2^ for 80 s twice/day for 6 days	Rat visual cortex primary neuronal cultures	TTX	NIR increased COX activity in TTX-treated neurons.
Wong-Riley et al. (2005)	LED	670 nm, 720 nm, 770 nm, 830 nm, 880 nm—singly or in combination at 50 mW/cm^2^, 80 s once/day	Rat visual cortex primary neuronal cultures	TTX / KCN	High dose KCN blocks NIR mediated increase in COX activity.
Liang et al. (2006b)	LED	670 nm at 50 mW/cm^2^ single treatment lasting 600 s	Rat visual cortex primary neurons cultures	KCN	NIR reduces apoptosis and ROS levels in treated neurons.
Ying et al. (2008)	LED	670 nm at 50 mW/cm^2^ for 80 s twice/day for 2 days	Rat visual cortex and striatal primary neuronal cultures	Rotenone, MPP+	NIR reduces cell death and increases ATP production in rotenone and MPP+-treated neurons.
Liang et al. (2008)	LED	670 nm at 50 mW/cm^2^, 80 s twice/day for 2 days	Rat visual cortex and striatal primary neuronal cultures	Rotenone, MPP+	NIR reduces ROS and NO levels in rotenone and MPP+-treated neurons.
Trimmer et al. (2009)	Laser	810 nm at 50 mW/cm^2^ for 40 s single treatment	PD Cybrid cell-derived neuronal cells / SHSY5Y neuroblastoma cells	—	Improved mitochondrial movement 2 h after NIR treatment in PD cybrid-derived neuronal cells.

*Abbreviations: COX, cytochrome C oxidase; KCN, potassium cyanide; LED, light emitting diode; MPP+, 1-methyl-4-phenylpyridinium; NIR, near infrared light; NO, nitric oxide; PD, Parkinson’s disease; ROS, reactive oxygen species; TTX, tetradotoxin*.

Several *in vivo* studies validated the efficacy of NIR PBM in PD animal models (Table 2). Oueslati et al. (2015) using an adeno-associated virus (AAV) rat model of PD, managed to demonstrate retardation of α-synuclein toxicity and alleviation of synucleinopathy-related motor deficits with PBM. Several studies also showed similar rescue of SNpc dopaminergic neurons in MPTP–treated mice following PBM treatment (Shaw et al., 2010; Johnstone et al., 2014; Reinhart et al., 2015). PBM using a combination of lights with varying wavelengths (670 nm and 810 nm) in the NIR range delivered synchronously or sequentially were also tested in rodents, with positive results (Reinhart et al., 2017). Encouraging results from these studies prompted experiments investigating the feasibility of delivering NIR from an intracranial source. This was first demonstrated successfully in two rodent studies by Benabid and colleagues (Moro et al., 2014; Reinhart et al., 2016). In the study conducted by Reinhart et al. (2016), an intracranial NIR delivery device was implanted in the vicinity of the SNpc of 6-hydroxydopamine (6-OHDA) lesioned Wistar rats, demonstrating the feasibility of stereotaxic device implantation into the ventral midbrain. Interestingly, in the experiments conducted by Moro et al. (2014) and Reinhart et al. (2016), pulsed delivery of NIR achieved better results than continuous delivery for the same dose of NIR treatment. Following these accomplishments, Darlot et al. (2016) were the first to report successful PBM treatment *via* an intracranially implanted NIR device in a monkey model of PD. This was followed by further experiments in nonhuman primate models exploring different dosage protocols, the extent of astrogliosis and long term safety of NIR using implanted devices (El Massri et al., 2016, 2017; Moro et al., 2016). With regards to non-invasive PBM treatments for PD, there are a few groups that have examined its efficacy in human subjects. Hamilton et al. (2019) and Santos et al. (2019) have recently reported slight improvements in motor and non-motor symptoms in PD patients after the administration of PBM using extracranial sources. In their trial, Santos et al. (2019) delivered PBM by alternating a 670 nm LED between the subjects’ left and right temples in 6 1-min blocks per session. Subjects were treated twice a week for a total of 9 weeks and demonstrated significant improvements in motor scores (Santos et al., 2019). In parallel, Hamilton et al. (2019) performed a study involving 6 PD subjects who adopted the practice of wearing of “PBM helmets” –buckets lined on the inside with NIR lights—regularly for 24 months and reported that treated subjects experienced substantial alleviation of both motor as well as non-motor symptoms. One postulation is that PBM targeted at cortical regions in PD can modulate dysfunctional neural circuits caused by a lack of dopamine, even if there is no direct action on SNpc dopaminergic neurons. If this is true, PBM delivered to cortical regions may be capable of symptom relief, but is unlikely to achieve any neuroprotective effect (Hamilton et al., 2019).

**Table 2:** *In vivo* studies in PD models.

Reference	Source	Treatment Parameters	Model	Effects
**Studies in drosophila fruitflies**				
Vos et al. (2013)	Laser	808 nm at 25 mW/cm^2^ single treatment for 100 s	PINK1 null mutant drosophila fruit flies	NIR rescues flight and mitochondrial defects and increases COX mediated respiration in PINK1 null mutant drosophila flies.
**Studies in rodents**				
Shaw et al. (2010)	External LED	670 nm at 40 mW/cm^2^, 90 s once/day for 2–4 days	Acute MPTP mouse model*	NIR reduced DA neuronal loss in SNpc and ZI in MPTP-treated mice.
Shaw et al. (2012)	External LED	670 nm at 40 mW/cm^2^ for 90 s once/day for: - 4 days (acute MPTP group); - 10 days (chronic MPTP group).	Acute MPTP mouse model; Chronic MPTP mouse model***	NIR reduces abnormal c-Fos expression in the STN and ZI of MPTP-treated mice.
Peoples et al. (2012)	External LED	670 nm at 40 mW/cm^2^ for 90 s once/day for: - 4 days (acute MPTP group); - 10 days (chronic MPTP group).	Acute MPTP mouse model; Chronic MPTP mouse model	NIR reduced retinal DA amacrine cell death in MPTP-treated mice.
Purushothuman et al. (2013)	External LED	670 nm at 4J/cm^2^ per day for 4 weeks	K3 transgenic mice^#^	NIR reduces ROS, hyperphosphorylated tau levels and DA neuronal loss in SNpc.
Johnstone et al. (2014)	External LED	670 nm at 50 mW/cm^2^ for 90 s twice/day for 2–4 days	Acute MPTP mouse model	NIR reduced SNpc DA neuronal loss in MPTP-treated mice in dose-dependent fashion.
Moro et al. (2014)	Intra-cranial LED implant	670 nm, 0.16 mW given as follows: - 90 s twice/day for 2 days; - continuously for 6 days.	Acute MPTP mouse model	NIR delivered by an intracranial device reduced DA neuronal loss in SNpc of MPTP-treated mice.
Reinhart et al. (2015)	External LED	810 nm, 160μW, 90 s twice a day for 2 days	Acute MPTP mouse model	NIR reduced DA neuronal loss in SNpc of MPTP treated mice.
El Massri et al. (2016)	External LED	670 nm at 5.3 mW/cm^2^ given as follows: - 90 s twice/day for 2 days (acute MPTP group); - 90 s once/day for 4–7 days (subacute MPTP group)	Acute MPTP mouse model; Subacute MPTP mouse model**	The neuroprotective effects of NIR are dose-dependent. NIR treatments reduce MPTP mediated astrogliosis.
Reinhart et al. (2016)	Intra-cranial LED implant	670 nm delivered in the following ways: - 90 s twice a day at 0.16 mW totaling 634 mJ; - Continuous delivery at 333 nW totaling 634 mJ; - Continuous delivery at 0.16 mW totaling 304J	Striatal 6-OHDA injected hemi-parkinsonian rat	Pulsed delivery of NIR *via* intracranial device achieved better effects than continuous delivery for the same total dosage.
Reinhart et al. (2017)	External LED	670 nm and/or 810 nm in different combinations at 30 mW for 90 s twice a day for 2 days	Acute MPTP mouse model	Combined application of 670 nm and 810 nm light together, either sequentially or concurrently, achieved better outcomes.
Ganeshan et al. (2019)	External LED	670 nm at 50 mW/cm^2^ for 90 s once/day for 2, 5 or 10 days at back or hindlimbs	Acute MPTP mouse model	Preconditioning with NIR at sites distant from the head mitigate neurotoxic effects of MPTP and caused transcriptomic changes in the brain of MPTP-treated mice.
**Studies in non-human primates**				
Darlot et al. (2016)	Intra-cranial laser implant	670 nm, 10 Mw in cycles of 5 s on/60 s off delivered: - Over 5 days (subacute MPTP group) - Over 7 days (extended subacute MPTP group)	Subacute MPTP monkey model^†^; extended subacute MPTP monkey model^‡^	NIR reduced DA neuronal loss in SNpc of MPTP-treated monkeys.
Moro et al. (2016)	Intra-cranial laser implant	670 nm, 10 mW in cycles of [5 s on, 60 s off] for 25 days	Subacute MPTP monkey model	Extended duration of NIR treatment is not as beneficial as shortterm treatment.

*Acute MPTP mouse model: 25 mg/kg/day of MPTP for 2 days in 8–10 week old BALBc mice. **Chronic MPTP mouse model: 20 mg/kg/day of MPTP in 10 doses over 5 weeks in 8–10 week old BALBc mice. ***Subacute MPTP mouse model: 25/mg/kg/day of MPTP for two consecutive days a week for 2 weeks in 8–10 week old BALBc mice. ^#^K3 transgenic mice: these mice harbor the pathogenic K369I mutation which leads to overexpression of hyperphosphorylated tau that causes progressive SNpc degeneration. ^†^Subacute MPTP monkey model: 1.5 mg/kg/day of MPTP for five consecutive days in adult male macaque monkeys. ^‡^Extended subacute MPTP monkey model: 2.1 mg/kg/day of MPTP for seven consecutive days in adult male macaque monkeys. Abbreviations: 6-OHDA, 6-hydroxydopamine ; COX, cytochrome C oxidase; DA, dopaminergic; LED, light emitting diode; MPTP, 1-methyl-4-phenyl-1,2,3,6-tetrahydropyridine; NIR, near infrared light; PD, Parkinson’s disease; ROS, reactive oxygen species; SNpc, substantia nigra pars compacta; STN, subthalamic nucleus; ZI, zona incerta.

## Barriers to Popularizing NIR as a Neuroprotective Modality for PD

**Figure 2:**
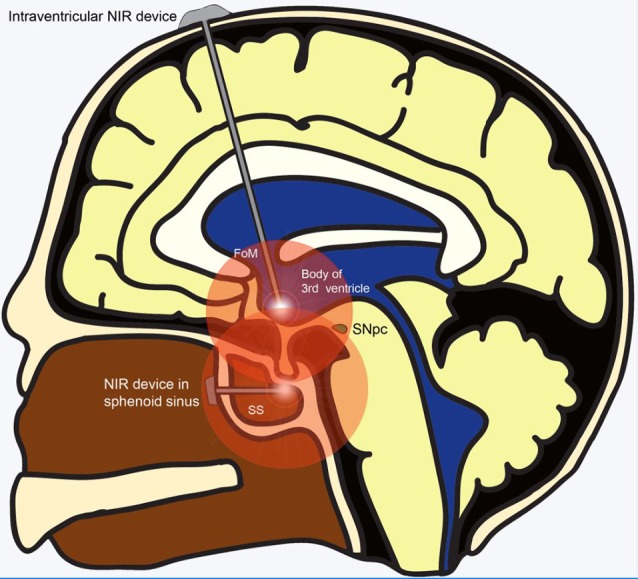
Alternative sites for implantation of NIR delivery device apart from the ventral midbrain. Wirelessly powered NIR delivery devices in the 3rd ventricle and the sphenoid sinus (SS), both of which are close to the ventral midbrain. Insertion of the device into the 3rd ventricle can be achieved safely *via* a straight trajectory traversing the Foramen of Monroe. Insertion of the NIR device within the SS in the posterior part of the nasal cavity can be achieved using an endoscopic endonasal approach. Abbreviations: FoM, foramen of Monroe; NIR, near-infrared; SNpc, substantia nigra pars compacta; SS, sphenoid sinus.

The development of an intracranially implanted NIR delivery device for PD is challenging for several reasons. First, based on their studies in mice brains, Moro et al. (2014) realized that the signal intensity of NIR decays at a rate of 65% per millimeter of brain tissue. In comparison with the brains of mice and small monkeys, the human brain is much larger and shielded by a thick bony calvarium. These barriers to light penetration, compounded by the fact that the midbrain SNpc is one of the most deep-seated parts of the human brain, present significant challenges for NIR delivery to the target region. Ultimately, this endeavor would involve the insertion of an LED *via* a safe trajectory deep into the ventral midbrain to effect NIR stimulation. Being our “seat of consciousness,” the midbrain is an exquisitely sensitive region of the human brain, with zero tolerance for any surgical mishap. Operating in such an important region of the brain inadvertently translates into greater risks for surgical candidates. Also, for neuroprotection to be effective, it has to be administered early in the course of the disease. Given the lack of effective early screening modalities, PD is largely diagnosed based on its clinical features which only manifest after 70%–80% of SNpc dopaminergic neurons have undergone degeneration. This implies that only 20%–30% of SNpc dopaminergic neurons remain available for salvage even if neuroprotective measures were implemented immediately at the time of diagnosis. Conversely, even if pre-clinical PD were able to be effectively screened for and diagnosed, the significant risks of surgical implantation may deter patients who are “seemingly well” from undertaking such a procedure, culminating in a poor uptake rate for this treatment modality. Perhaps safer regions for intracranial implantation of the LED could be explored, such as placement of the NIR LED just rostral to the midbrain tegmentum within the body of the third ventricle. This can be achieved safely under direct vision with the guidance of a neuro-endoscope and a carefully planned trajectory (Figure 2). Another site that could be considered for implantation is within the sphenoid sinus (SS) located in the posterior nasal space given its proximity to the ventral midbrain (Figure 2). Nevertheless, although near the upper brainstem, the SS remains separated from it by the bony clivus and the dura mater, which impedes the transmission of the NIR signal. Hence, simulation studies looking at the penetration of NIR through these layers of tissue would be required before commissioning the SS as a satisfactory location for LED placement. Second, given that PD runs its course over years, another challenge for such a device is the sustained delivery of NIR stimulation over a prolonged period. The presence of an efficient power source is imperative for such an undertaking. Existing batteries for intracranial implantable devices often require implantation in a subcutaneous pocket over the chest, with wires tunneled under the skin to connect it with the device’s intracranial portion. These device components are subject to the risks of breakage and microbial infection, which significantly increase device-related morbidity. The advent of miniaturized wirelessly powered devices may be able to overcome this technological limitation (Montgomery et al., 2015). Recent evidence advocating early DBS for PD (EARLYSTIM Trial, 2013) may be used to promote the use of NIR devices as co-implantation of NIR and DBS devices could be performed in the same setting (Schuepbach et al., 2013).

## Concluding Remarks

The idea of matchmaking NIR PBM—a mitochondrial modulatory instrument, with PD—a disease of bioenergetic failure, remains an attractive proposition. However, before getting mired in the race towards clinical translation, a step back to examine the gaps in our understanding of PBM seems worthy of our efforts. It seems that the mechanisms through which PBM augments mitochondrial function remain unclear. Information on its effects on gene transcription, mitochondrial dynamics and immunomodulation are also scant and limited. These shortfalls are areas that we believe future research efforts can be directed at. A further concern of intracranial NIR delivery surrounds the need for device implantation into the midbrain. Given that manipulation in this treacherous region of the brain could potentially lead to devastating clinical deficits it would not be unreasonable to anticipate that such a treatment modality would be met with resistance from patients with early PD. Alternative sites for implantation of the NIR delivery device, such as the intranasally or intraventricularly, could potentially be explored. We hope that technological advancements in the future can overcome these shortcomings and produce a device optimized for use in human subjects.

## Author Contributions

AF wrote the first draft in consultation with all other co-authors, who provided inputs. K-LL proof-read and completed the final draft. TS and TY edited the manuscript and provided expert advice and intellectual inputs.

## Conflict of Interest

The authors declare that the research was conducted in the absence of any commercial or financial relationships that could be construed as a potential conflict of interest.
